# Role of KCNB1 in the prognosis of gliomas and autophagy modulation

**DOI:** 10.1038/s41598-017-00045-7

**Published:** 2017-02-08

**Authors:** Hao-Yuan Wang, Wen Wang, Yan-Wei Liu, Ming-Yang Li, Ting-Yu Liang, Ji-Ye Li, Hui-Min Hu, Yang Lu, Chen Yao, Yong-Yi Ye, Yong-Zhi Wang, Shi-Zhong Zhang

**Affiliations:** 1 Department of Neurosurgery, Zhujiang Hospital, Southern Medical University, Guangzhou, China; 2 The National Key Clinical Specialty, The Engineering Technology Research Center of Education Ministry of China Guangdong Provincial Key Laboratory on Brain Function Repair and Regeneration, Department of Neurosurgery, Zhujiang Hospital, Southern Medical University, Guangzhou, China; 3 Beijing Neurosurgical Institute, Capital Medical University, Beijing, China; 4 Chinese Glioma Cooperative Group (CGCG), Beijing, China; 5 Department of Neurosurgery, The Second Affiliated Hospital of Soochow University, Suzhou, China; 6 Department of Neurosurgery, Beijing Tiantan Hospital, Capital Medical University, Beijing, China; 7 Department of Radiation Therapy, Beijing Tiantan Hospital, Capital Medical University, Beijing, China

## Abstract

Increasing evidence suggests that ion channel genes play an important role in the progression of gliomas. However, the mechanisms by which ion channel genes influence the progression of glioma are not fully understood. We identified KCNB1 as a novel ion gene, associated with malignant progression and favorable overall survival (OS) and progression-free survival (PFS) in glioma patients from three datasets (CGGA, GSE16011 and REMBRANDT). Moreover, we characterized a novel function of autophagy induction accompanied by increased apoptosis and reduced proliferation and invasion of glioma cells for KCNB1. KEGG pathway analysis and *in vitro* studies suggested that the ERK pathway is involved in KCNB1-mediated regulation of autophagy, which was confirmed by inhibition of KCNB1-induced autophagy by using a selective ERK1/2 inhibitor (U0126) or siERK1/2. *In vivo* studies showed that KCNB1 induced autophagy while inhibiting tumor growth and increasing survival. Overall, our studies define KCNB1 as a novel prognostic factor for gliomas that exerts its tumor suppressive function through autophagy induction.

## Introduction

Gliomas are the most common primary tumors of the central nervous system and patients with grade IV glioma, or glioblastoma multiforme (GBM) have a median survival times of only 12.2 to 18.2 months^1, 2^. The current standard of therapy is surgery, followed by concurrent radiation and temozolomide administration. Despite the continuous progress in neurosurgery, the infiltrative behavior of gliomas precludes complete tumor resection and is certainly the primary reason for poor clinical outcome for patients^3, 4^. In recent years, the role of ion channels in glioma has received wide attention^5^. For example, *NKCC1* is constitutively expressed in gliomas, and its expression positively correlates with invasiveness^6^; Both KCa1.1 and KCa3.1 have an important role in glioma cell migration^7, 8^.

Numerous studies have reported that Kv, the largest subset of potassium channels gated by changes in the membrane potential, is associated with several cancers. For example, low levels of *KCNA1* correlate with increased aggressiveness of primary breast tumors^9^; repression of *KCNA5* plays a role in Ewing sarcoma and neuroblastoma^10^; blocking the *KCNK9* channel inhibits tumor growth and metastasis^11^; Kir2.2 was overexpressed in human cancer^12^; high Kv1.3 and Kv1.5 expression levels are markers of breast, colon, and prostate cancer^13^; overexpression of Kv1.1 is detected in medulloblastoma^14^; elevated Kv11.1 expression indicates blood cancer^15^. *KCNB1* (Kv2.1), the principal voltage-gated potassium channel (Kv) channel underlying delayed-rectifier currents (IDR) in most mammalian brain neurons, regulates excitability during periods of high frequency firing^16, 17^. Although several studies have demonstrated that regulation of *KCNB1* is involved in neuronal apoptosis^18, 19^ and *KCNB1* mutation can result in early epileptic encephalopathy^20^, the role of *KCNB1* in gliomas remains unknown.

In this study, we report for the first time that *KCNB1* is associated with malignant progression and outcome in gliomas using three datasets (CGGA, GSE16011 and REMBRANDT). Further, biological and functional analyses suggest that *KCNB1* affects the autophagy induction by regulating the ERK pathway, which may provide mechanistic insights into the aggressiveness of gliomas and contribute to the development of new therapeutic approaches.

## Materials and Methods

### Patients and samples

All glioma samples included in our study were from the Chinese Glioma Genome Atlas (CGGA), including 109 grade II gliomas, 41 grade III gliomas and 83 primary GBM. The patients underwent surgical resection between January 2006 and December 2009. Patients were eligible for the study if the diagnosis of glioma was established histologically according to the 2007 WHO classification. These patients underwent surgery and were followed-up at the Beijing Tiantan hospitals. Clinicopathological data, including gender, age, pathologic diagnosis and the results of molecular analysis, were obtained. All patients provided written informed consent for research purposes, according to guidelines approved by the institutional Review Board of Ethics at the Southern Medical University and Capital Medical University. The experimental protocols of all experiments involving human were approved by the ethical committee of Southern Medical University and performed in accordance with approved guidelines and regulations.

Whole transcriptome sequences of 233 gliomas were obtained from the Chinese Glioma Genome Atlas (CGGA) database (http://www.cgga.org.cn)^21^. The other two whole-genome mRNA expression microarray datasets were downloaded from the repository for molecular brain neoplasia data (REMBRANDT, http://caintegrator.nci.nih.gov/rembrandt) and the GSE16011 dataset (http://www.ncbi.nlm.nih.gov/geo/query/acc.cgi?acc=GSE16011).

### Cell lines and construction of cells stably expressing *KCNB1*

U87MG and U118MG cells were cultured in DMEM (Gibco) with 10% fetal bovine serum (Gibco), and incubated at 37 °C in a humidified incubator. U87MG and U118MG cells were converted into *KCNB1* overexpressing cells (*KCNB1*+) by stable transfection. A pEX-Lv201/eGFP plasmid was constructed by ligating the PCR-amplified eGFP into the KpnI and XhoI sites of the pEX-Lv201 vector (GeneCopoeia Inc.). Control cells were transduced with a pEX-Lv201/eGFP vector. The pEX-Lv201/*KCNB1*-eGFP construct was generated by cloning the PCR-amplified *KCNB1* into the KpnI and XhoI sites of the pEX-Lv201/eGFP construct (Supplementary Figure S1). Lentivirus carrying plasmid was added to the culture medium. The medium was changed after 24 h. After 72 h, stable cells overexpressing *KCNB1* or empty vector were selected during three days of puromycin (2 μg/ml) treatment.

### Chemicals and antibodies

U0126 was purchased from APExBIO Technology LLC (A1337, APExBIO). anti-LC3B (catalogue: 3868S), anti-phospho ERK1/2 (4370), anti-ERK1/2 (9102), anti-phospho JNK1/2 (4668), anti-JNK1/2 (9252), anti-phospho p38 (4511), anti-p38 (8690) were obtained from Cell Signaling Technology. Anti-*KCNB1* was obtained from abcam (catalogue: ab106513, Abcam, 1:1000).

### Proliferation, migration and invasion assays

For the migration assay the cells were plated at a density of 2 × 10^5^ cells/well onto six well plates and artificial wound tracks were created by scraping with a specific scratcher within the confluent monolayers after 24 hours. Upon removal of the detached cells by PBS washing, the medium was refreshed and the ability of the cells to migrate into the wound area was assessed by comparing the pixels of the wound tracks in the images taken at the beginning of the exposure (time 0), with those taken after 6, 12, 18 and 24 hours.

For the MTT assays, the cells were grown to exponential phase and detached by trypsin treatment. A total of 2000 cells/well were plated onto 96-well tissue culture plates (100 μl complete medium/well) and cultured at 37 °C in 5% CO2. 20 μL per well of MTT reagent was added and incubated at 37 °C for 1 hour. Subsequently, the absorbance values of each well were measured with a microplate spectrophotometer at 490 nm after 0, 24, 48,72, 96 and 120 hours. The results were plotted as means ± SD of three independent experiments.

### Evaluation of apoptosis and autophagy induction

For the apoptosis assay, the cells were harvested and washed with cold PBS, and then the cells were stained first with 5 μl Annexin V/Alexa Fluor 647 and 10 μl 20 ug/ml PI (Beijing 4A Biotech Co., Ltd, China) for 15 min at 4 °C in the dark. The cells were then analyzed using an ImageStreamX Mark II instrument. The results were analyzed and displayed using IDEA software.

For the electronic microscopy studies, cells were washed twice with PBS and fixed with a glutaraldehyde fixative (2.5% glutaraldehyde, 2% paraformaldehyde) for 2 hours at 4 °C, and then postfixed in 1% Osmic acid for 2 hours at 4 °C. The samples were dehydrated in a graded series of ethanol, transferred to propylene epoxide and embedded in Epon-812 resin. In all, 50 nm sections were cut and stained with uranyl acetate and lead citrate. Sections were examined using H-7650 (Leica, Japan) at 80 kv and photographed with an AMT CCD camera. Autophagic vacuoles were quantified for U87MG-*KCNB1*+ cells (n = 50 cells) and U87MG-CTR cells (n = 50 cells).

### DAVID analysis of associated genes in gliomas

Significant analysis of microarray (SAM) was performed in gliomas to identify differentially expressed genes, followed by GO and KEGG Pathway analysis of those genes highly expressed in the high-*KCNB1*group, performed using DAVID^22^ for function annotation.

### RNA isolation and qRT-PCR

All tissue samples were immediately snap-frozen in liquid nitrogen after surgery. Total RNA from frozen tumor samples was extracted using the mirVana miRNA Isolation Kit (Ambion) according to the manufacturer’s protocol. RNA concentration and quality were measured using the NanoDrop ND-1000 spectrophotometer (NanoDrop Technologies). cDNA was synthesized by M-MLV (Moloney murine leukemia virus) reverse transcriptase (Invitrogen) from 2 μg of total RNA. Oligo (dT) 18 was used as the primer for reverse transcription of mRNA. Quantitative real-time RT-PCR was carried out in a 7500 real-time PCR System (Applied Biosystems) using the SYBR Select Master Mix (Applied Biosystems) according to the manufacturer’s instructions. The real-time PCR primers were as follows: *KCNB1* forward-5′CCATTCTGCCATACTATGTCACC-3′, reverse-5′AGCAAGCCCAACTCATTGTAG-3′. GAPDH forward-5′CCACCCATGGCAAATTCCATGGCA-3′, reverse-5′TCTAGACGGCAGGTCAGGTCCACC-3′. GAPDH was used as an internal control, and fold changes were calculated by relative quantification (2^−ΔΔCt^).

### Small interfering RNA (siRNA) transfection

Cells were seeded into 6-well plates at a density of 2 × 10^5^ cells per well and allowed to reach approximately 70% confluence on the day of transfection. Cells were transfected with 30 nM siRNA using Lipofectamine 2000 transfection reagent (Life Technologies) as described in the manufacturer’s protocol. siRNA against ERK1/2 (siERK) and siRNA against *KCNB1* (si*KCNB1*) were obtained from GenePharma (Shanghai GenePharma Co., Ltd).

### Western blot and immunofluorescence for cultured cells

Whole-cell lysates were prepared using RIPA buffer (Cell Signal Technology). Western blot analysis was performed as previously described^23^. Immunofluorescence staining was performed on cells as previously described^23^ and then monitored with a fluorescence microscope.

### *In vivo* studies

The mouse model was generated via subcutaneous or intracranial injection of U87MG-*KCNB1*+ or U87MG-CTR cells in female nude mice (age 6 weeks). Mice were housed in specific pathogen-free conditions. For subcutaneous xenografts, 1 × 10^6^ cells, suspended in 100 ul of PBS, were injected subcutaneous into the flank of five nude mice per group. Tumor length, width and weights were measured weekly and the volume was calculated according to the formula (length × width^2^)/2. For orthotopic xenografts, a Hamilton syringe and microinfusion syringe pump (0.5 ml/min) were used to implant 1 × 10^6^ cells into the brain of 5 nude mice per group simultaneously. The sizes of tumors were monitored by MRI after 4 weeks. Upon detection of obvious declining health, the mice were killed and their brains were collected and submitted for histological examination. All experiments were performed in accordance with the Institutional Animal Care and Use Committee of Southern Medical University. All experimental protocol involving mice were approved by the ethical committee of Southern Medical University and performed in accordance with approved guidelines and regulations.

### Statistical Analysis

All experiments were performed three times and the results are shown as the mean ± standard deviation. Statistical analysis was performed using Graphpad Prism 5.0 by Student’s t test or the Mann–Whitney test. The associations between *KCNB1* expression and clinicopathological features were tested by Pearson Chi-Square test. Kaplan–Meier and log-rank methods were used to compare OS and PFS curves using SPSS version 20. Statistically significant variables in the univariable analysis were included in multivariable analysis using Cox proportional hazards model. All statistical tests were two-sided. A difference was considered significant when p < 0.05.

## Results

### Integrated analysis of the whole-genome identifies a negative correlation of *KCNB1* with malignant progression in gliomas

**Figure 1:**
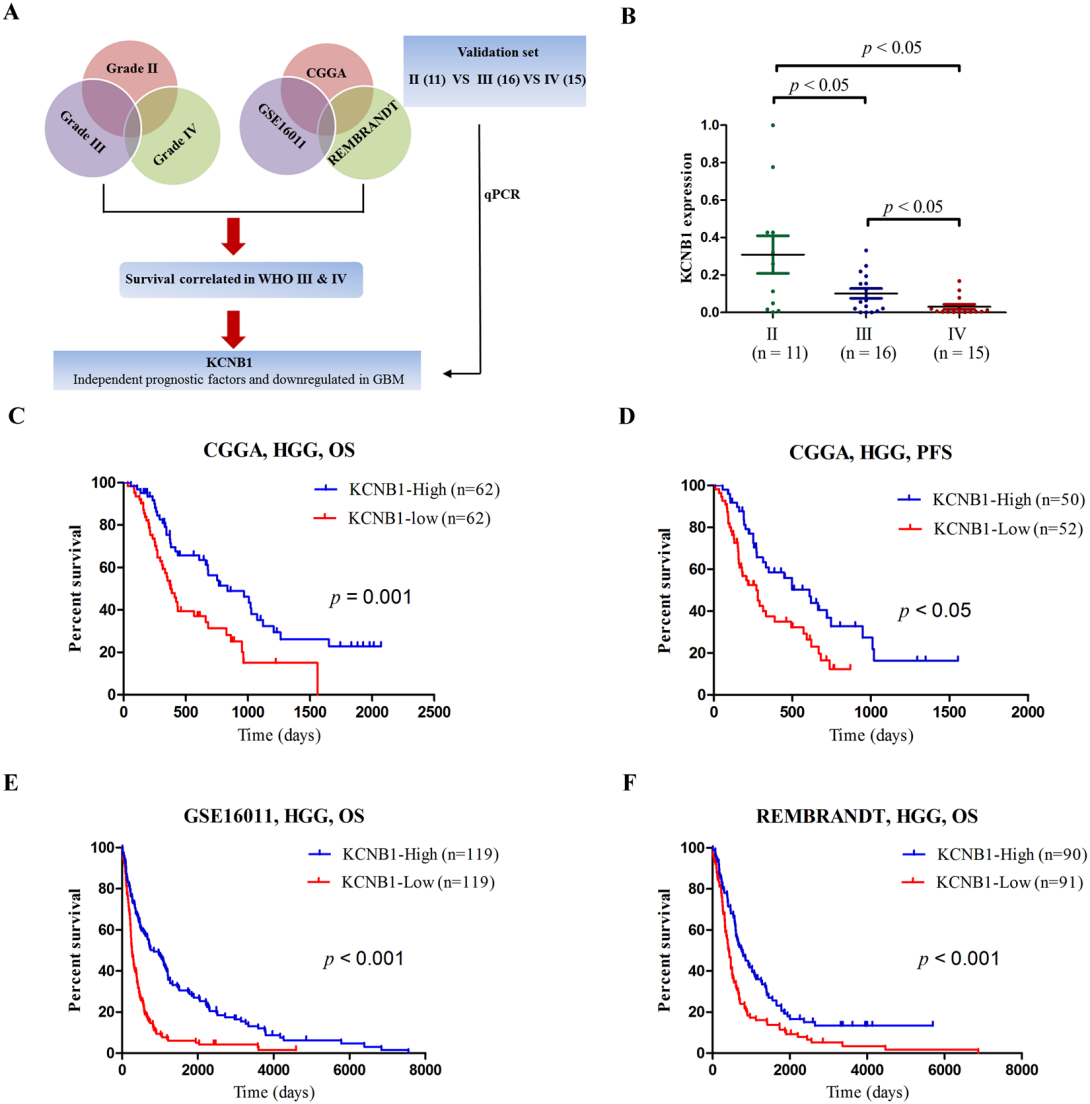
Discovery of a prognostic ion gene in gliomas. (**A**) Schematic strategy used to identify a prognostic ion gene in gliomas. (**B**) The qRT-PCR analysis of relative KCNB1 expression in glioma tissue samples from the validation cohort. *P* values were calculated with two-sided Student’s t test. (**C**,**D**) Kaplan–Meier OS and PFS analysis of patients with high grade (83 patients with primary GBM and 41 patients with grade III) in CGGA. Survival among KCNB1-high group (n = 62) and KCNB1-low group (n = 62) patients is shown. *P* values were calculated using the two-sided log-rank test. (**E**,**F**) Kaplan–Meier OS analysis of patients with high grade in GSE16011 (154 patients with grade IV and 84 patients with grade III) and REMBRANDT (110 patients with grade IV and 71 patients with grade III). OS, overall survival; PFS, progression-free survival. *P* values were calculated with a two-sided log-rank test.

A total of 280 ion channel genes were evaluated for the present study (Supplementary: Table S1). To identify ion channel genes associated with grade progression, we first compared genome expression between grade II, III and IV gliomas in three datasets (CGGA, GSE16011, and REMBRANDT), followed by a two-sided log-rank test performed to analyze each ion gene in high grade (III + IV) gliomas (Fig. 1A). The ion channel genes were finally validated in an independent set of glioma tissue samples by performing qRT-PCR (Fig. 1B). The results showed that *KCNB1* was the only ion channel gene that correlated with malignant progression in gliomas.

### Association of *KCNB1* mRNA and protein expression with outcome

To identify whether *KCNB1* can be used as a prognostic biomarker, we correlated *KCNB1* with overall survival (OS) and progression-free survival (PFS) in samples from 83 patients with primary GBM and 41 patients with grade III glioma who underwent tumor treatment in our cohort. We defined the *KCNB1*-high and *KCNB1*-low groups according to the median level of *KCNB1* expression. The *KCNB1*-high group was associated with favorable OS (median OS = 27.9 months, 95% confidence interval [CI] = 15.8 to 39.9 months; vs median OS = 12.5 months, 95% CI = 9.7 to 15.3 months; hazard ratio [HR] = 2.2, 95% CI = 1.35 to 3.5; *p* < 0.001, two-sided log-rank test, Fig. 1C) and PFS (median PFS = 20.3 months, 95% confidence interval [CI] = 12.9 to 27.6 months; vs median PFS = 9.3 months, 95% CI = 5.7 to 13.0 months; hazard ratio [HR] = 2.0, 95% CI = 1.2 to 3.3; *p* < 0.05, two-sided log-rank test, Fig. 1D). To control the influence of age at diagnosis, gender, chemotherapy, and radiotherapy on the stratification of gliomas, we used the Cox regression model as shown in Table 1. The prognostic value of *KCNB1* expression was still significant and was independent of other clinical characteristics. The prognostic value of *KCNB1* was validated using the GSE16011 and REMBRANDT datasets (Fig. 1E,F).

**Table 1:** Cox proportional hazards regression analyses of KCNB1 expression and other characteristics in relation to overall survival in gliomas from the CGGA cohort.

Clinical Characteristic	No. (%)	OS months (95% CI)	Univariate analysis	Multivariable analysis		
			*P*	HR	95%CI	*P*
Age, years			0.005			0.4
≤50	62 (50%)	22.5–40.9				
>50	62 (50%)	10.9–13.9				
Gender			0.297			
Male	79 (63.7%)	4.9–24.1				
Female	45 (36.3%)	11.2–34.2				
Chemotherapy			0.002	0.43	0.26–0.72	0.001
positive	78 (62.9%)	17.9–37.2				
negative	32 (25.8%)	7.9–11.4				
NA	14 (11.3%)					
Radiotherapy			0.07			
positive	91 (73.4%)	10.2–35.0				
negative	21 (16.9%)	1.2–19.7				
NA	12 (9.7%)					
KCNB1			0.001	0.45	0.25–0.80	0.007
High expression	62 (50%)	15.8–40.0				
Low expression	62 (50%)	9.7–15.3				

### Effects of *KCNB1* on apoptosis, autophagy, cell growth and invasion

**Figure 2:**
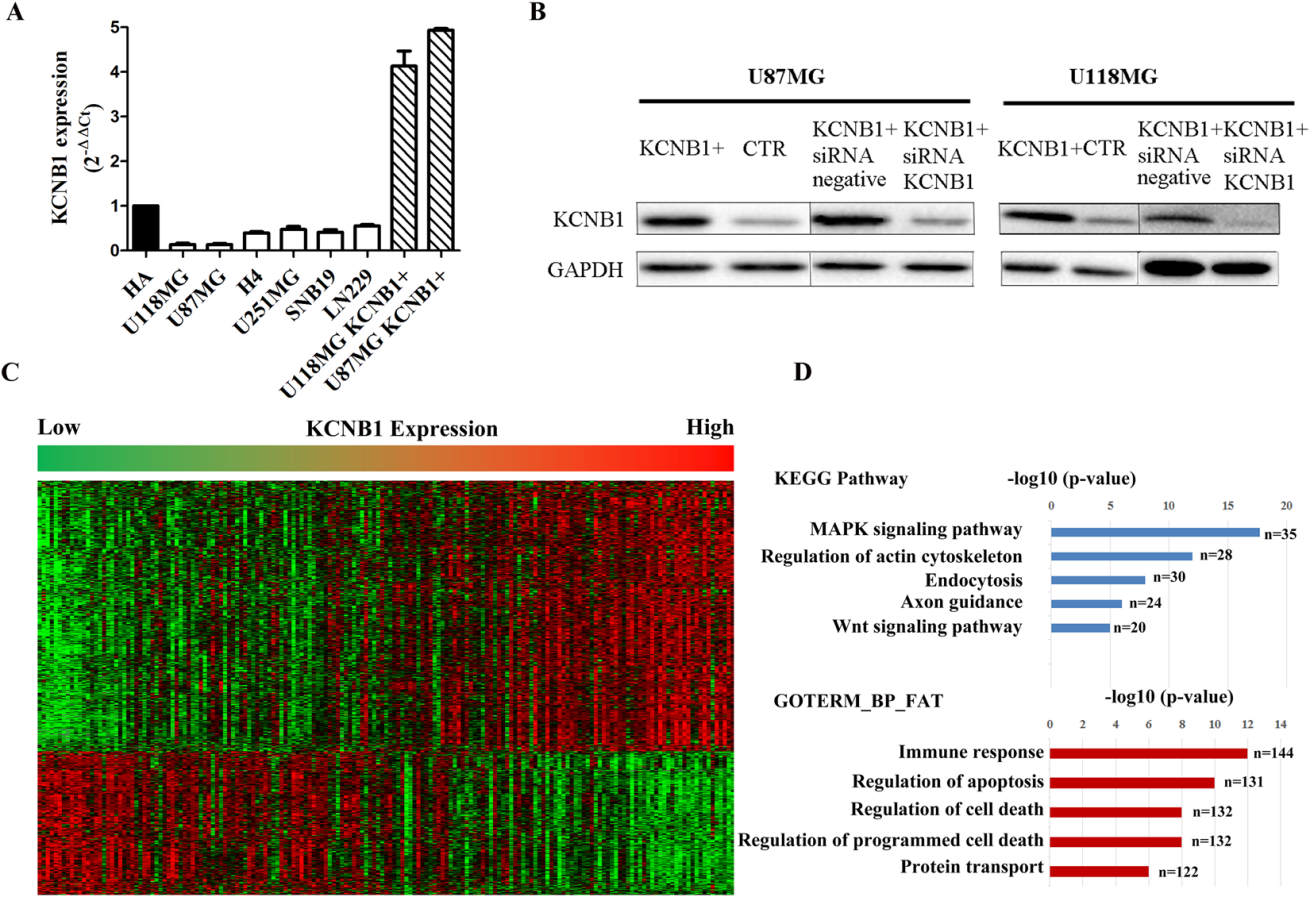
KCNB1 mRNA and protein expression in glioma cells and Clustering analysis of whole transcriptome sequencing from CGGA. (**A**) KCNB1 mRNA expression in glioma cells (vs. normal human Astrocytes cells HA) and cells transduced with the KCNB1 vector (KCNB1+). (**B**) Western blot of KCNB1+ cells. (**C**) Patterns of whole transcriptome sequencing were associated with KCNB1 in gliomas using one-dimensional hierarchical clustering analysis. (**D**) Functional enrichment analysis of associated genes, indicating the functional roles of gene sets in different subgroups. Enrichment results for biological processes were obtained from the GO and KEGG Pathway databases.

To study the biological role of *KCNB1*, we examined its mRNA expression and observed lower expression levels in all malignant cells compared to non-tumorigenic cells (Fig. 2A). U87MG and U118MG cells were selected for further investigation because they expressed the lowest levels of *KCNB1* mRNA. We successfully established *KCNB1* overexpressing subclones and empty vectors with more than 90% efficiency in each cell type (Supplementary Figure 2A). Furthermore, quantitative reverse-transcription polymerase chain reaction demonstrated a 47-fold and 38-fold increase of *KCNB1* mRNA expression in U87MG-*KCNB1*+ and U118MG-*KCNB1*+, respectively (Fig. 2A). Western blot analysis also showed an increase in *KCNB1* protein expression in U87MG-*KCNB1*+ and U118MG-*KCNB1*+ cells (Fig. 2B).

Significant analysis of microarray (SAM) was performed on the data from high-grade gliomas (III + IV) in CGGA to identify differentially expressed genes (Fig. 2C). This was followed by GO and KEGG pathway analysis of the differentially expressed genes in the *KCNB1*-high expression group using DAVID^22^ for functional annotation. The top five GO terms showed that *KCNB1* was associated with immune response, regulation of apoptosis, protein transport, regulation of cell death and regulation of programmed cell death (Fig. 2D). KEGG pathway analysis showed that the MAPK signaling pathway was the predominant cellular pathway associated with *KCNB1* expression (Fig. 2D).

**Figure 3:**
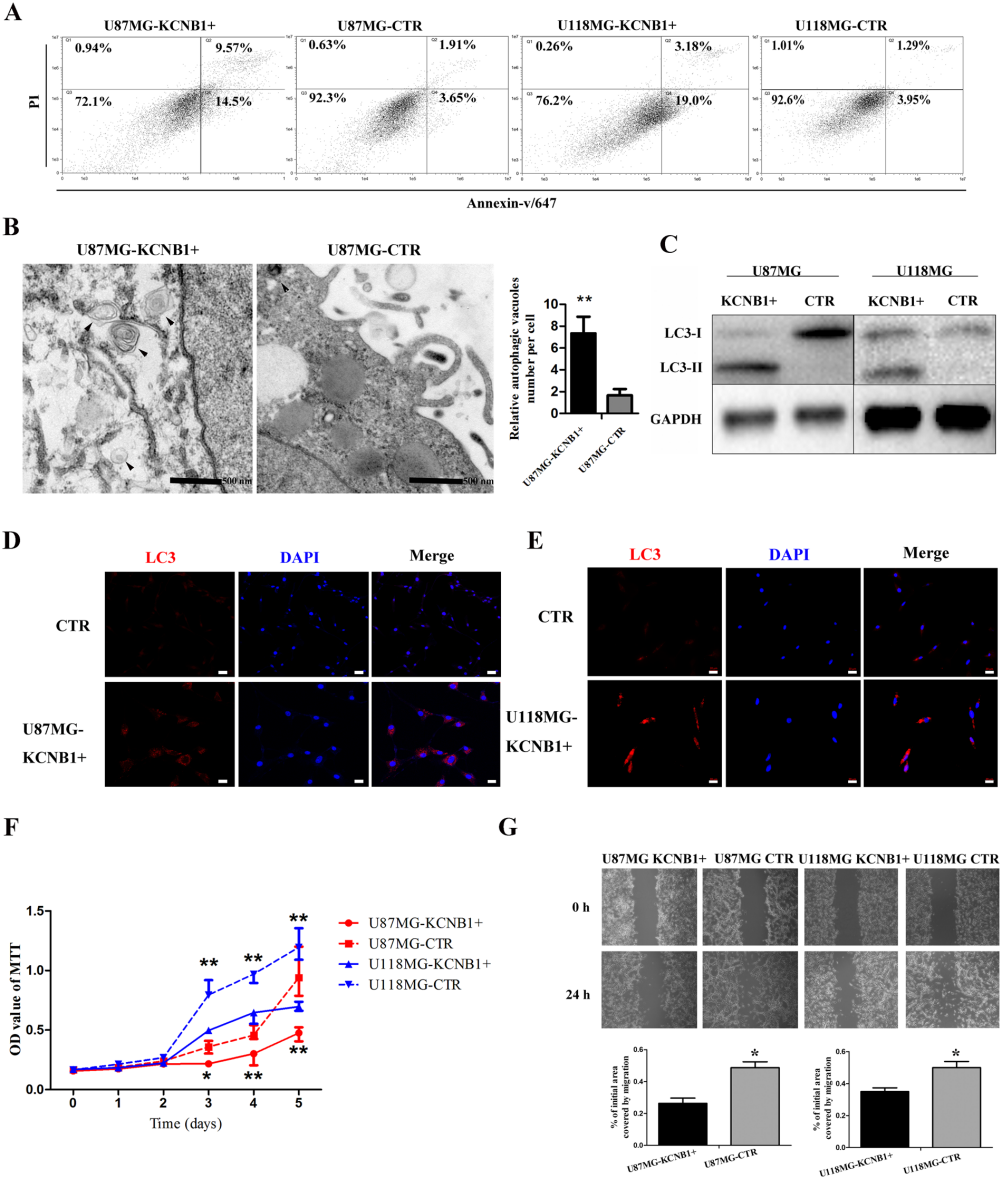
The KCNB1 overexpression affected the apoptosis, autophagy, proliferation and migration of glioma cells. (**A**) Flow cytometry analysis of apoptosis as detected by Annexin V and PI levels in the U87MG and U118MG cells (Q2: late apoptosis; Q4: early apoptosis). (**B**) Electron microscopy phenotypes in U87MG-KCNB1+ cells, showing increased autophagic vacuoles near the nucleus compared with U87MG-CTR cells. (**C**) Western blot showing the increased expression of LC3-II in U87MG-KCNB1+ and U118MG-KCNB1+ cells. (**D**,**E**) Immunofluorescence staining showing increased autophagic vacuoles (LC3) in U87MG-KCNB1+ and U118MG-KCNB1+ cells. Scale bar, 20 um. (**F**) Growth curves of U87MG and U118MG cells (KCNB1+ vs. CTR). Cell viability was assessed by MTT assay. Data shown are the mean ± SD (n = 3). (**G**) Wound-healing assay in U87MG and U118MG cells (KCNB1+ vs CTR). The wound gaps were photographed and measured. *P* values were calculated with two-sided Student’s t test. *p < 0.05, **p < 0.01.

As GO analysis showed that *KCNB1* had a tight association with apoptosis, we performed Annexin-V staining in U87MG-*KCNB1*+ and U118MG-*KCNB1*+ cells. The results showed a statistically significant increase in apoptosis in U87MG-*KCNB1*+ and U118MG-*KCNB1*+ cells, as shown in Fig. 3A. U87MG-*KCNB1*+ cells showed an increase in early apoptosis from 3.65% to 14.5% and late-apoptosis from 1.91% to 9.57% compared to control cells. U118MG- *KCNB1*+ cells showed an increase in early apoptosis from 3.95% to 19.0% and late-apoptosis from 1.29% to 3.18% compared to control cells. Many stimuli cause apoptosis also trigger autophagy and KEGG pathways analysis showed that *KCNB1* was associated with MAPK signaling pathway, which can control the balance of apoptosis and autophagy^24, 25^. Thus we inferred that *KCNB1* also had a tight association with autophagy. Immunoblot and immunostaining for LC3 revealed increased autophagy in U87MG-*KCNB1*+ and U118MG-*KCNB1*+ cells compared with U87MG-CTR and U118MG-CTR cells (Fig. 3C–E). Additionally, scanning electron microscopic showed varied morphological appearances of *KCNB1*+ cells including rounded shape and a significant increase in cytoplasmic vacuolization. Quantitative analysis confirmed an increase in autophagic vacuoles in *KCNB1*+ cells compared to CTR cells (Fig. 3B). These *KCNB1*+ subclones showed a reduced rate of cell proliferation and wound healing (Fig. 3F,G), indicating that *KCNB1* expression leads to both growth inhibition and attenuated cell migration. *KCNB1* small interfering mRNA transfection of U87MG-*KCNB1*+ and U118MG-*KCNB1*+ cells restored wound healing (Supplementary Figure 2B).

### *KCNB1* regulates autophagy via the ERK pathway

**Figure 4:**
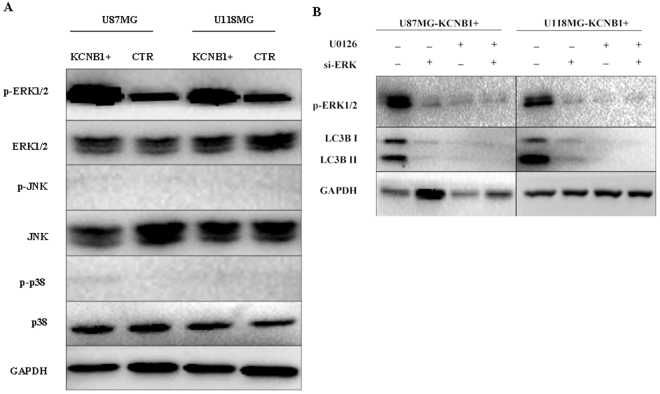
The KCNB1 overexpression induces autophagy via ERK pathway. (**A**) The expression level of phospho-ERK1/2, ERK1/2, phospho-JNK1/2, JNK1/2, phospho-p38, and p38 were detected by western blot analysis in U87MG and U118MG cells (KCNB1+ vs CTR). (**B**) Western blot analyses of phospho-ERK1/2 and LC3-II were performed in KCNB1+ cells treated by U0126 or si-ERK.

KEGG pathway analysis showed that *KCNB1* was associated with the MAPK signaling pathway (Fig. 2D). Mitogen activated protein kinases (MAPKs), including ERK, c-Jun N-terminal kinase (JNK), and p38, are the critical kinases that play an important roles in a variety of biological processes, such as cell proliferation, differentiation, apoptosis, and autophagy^26–28^. To determine the roles of these kinases in *KCNB1*-induced autophagy, we evaluated the phosphorylation status of these MAPK proteins in U87MG and U118MG cells. The level of phosphorylated ERK1/2 was significantly increased in U87MG and U118MG cells with *KCNB1* overexpression, but no significant changes were observed in the level of phosphorylated JNK1/2 and phosphorylated p38 in these cells (Fig. 4A). To further validate of the role of ERK signaling in U87MG and U118MG cells with *KCNB1* overexpression, U0126 (specific inhibitors of ERK1/2) was used to determine whether the inhibition of the ERK pathway affected the level of autophagy. Not only did pretreatment with U0126 inhibit the *KCNB1* induced ERK1/2 phosphorylation, but it also decreased the level of LC3II conversion (Fig. 4B). Moreover, siRNA-mediated knockdown of ERK1/2 resulted in a significant attenuation of *KCNB1*-induced LC3II conversion (Fig. 4B). These results suggested that the ERK pathway is involved in *KCNB1*-mediated regulation of autophagy.

### Effects of *KCNB1 in vivo*

**Figure 5:**
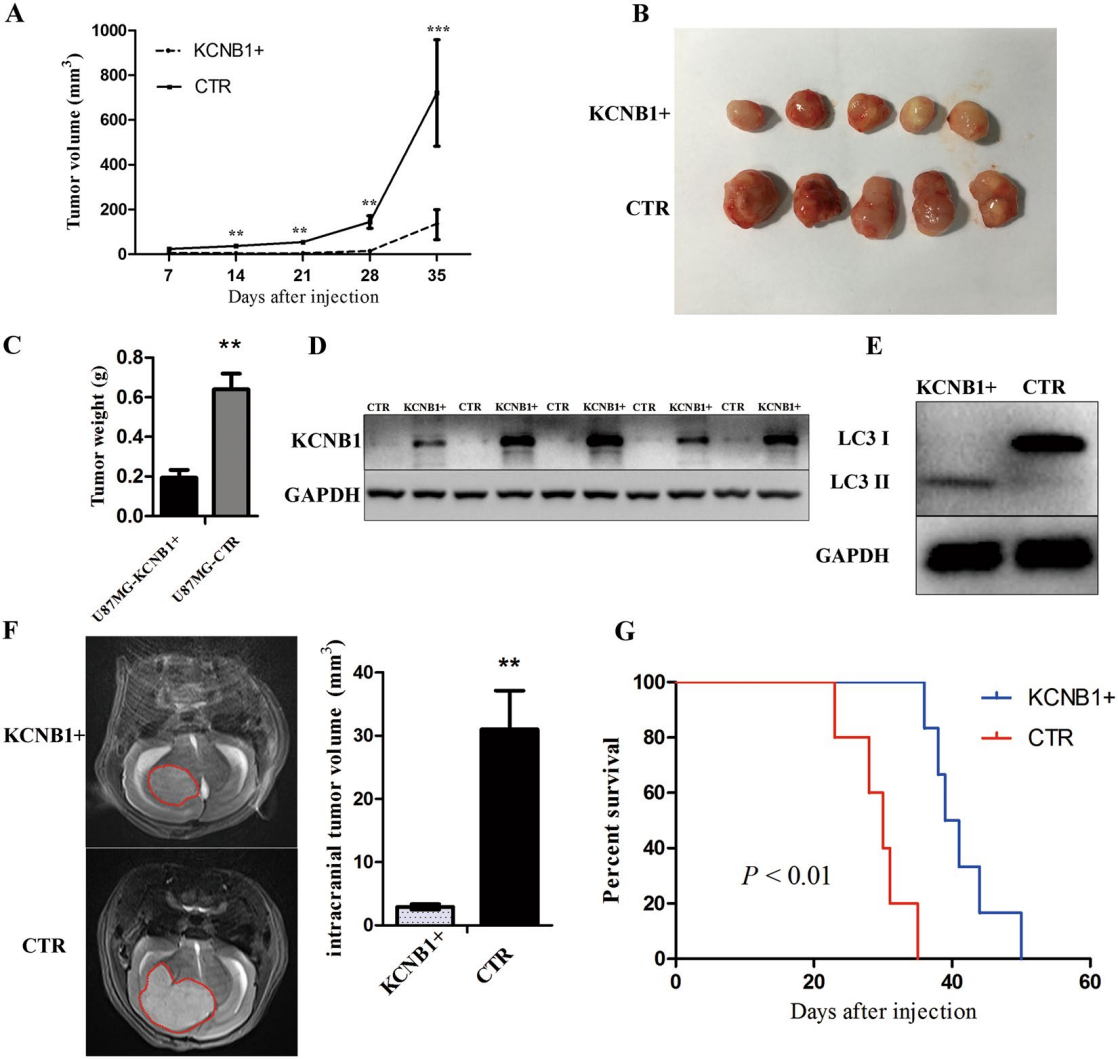
Effects of KCNB1 *in vivo*. (**A**) Tumor volumes were measured every seven days. Mean tumor volume was calculated. The results are presented as the mean ± SD. (**B**) A xenograft model consisting of nude mice with U87MG-KCNB1+ or U87MG-CTR cells injected subcutaneously into the 6-week-old mice (n = 5/group). KCNB1 overexpression impairs subcutaneous xenograft growth of glioma cells. (**C**) The tumor weight was measured for each xenograft. (**D**) KCNB1 protein levels in U87-KCNB1+ tumor tissues (n = 5) and U87-CTR tumor tissues (n = 5) were analyzed by western blotting. (**E**) Western blot analysis showing the overexpression of LC3-II in lysates from frozen tissues from the U87MG-KCNB1+ mice compared with the U87MG-CTR mice. (**F**) The sizes of orthotopic brain tumor xenografts were monitored by MRI after 4 weeks. KCNB1 overexpression in U87MG cells impairs tumorigenesis upon orthotopic injection. n = 5 mice per group. (**G**) Survival curves in the two groups of mice. Statistically significant differences were determined by two-sided log-rank test. *p < 0.05, **p < 0.01, ***p < 0.001.

To investigate the role of *KCNB1* in tumorigenesis, we employed an *in vivo* model by subcutaneously injecting U87MG-*KCNB1*+ or U87MG-CTR cells into nude mice. The length and width of tumors were measured when the xenografts were visible at 14 days after injection, and the volumes of the tumors were calculated. As shown in Fig. 5A,B, the size of xenografts in the U87MG-*KCNB1*+ group was smaller than that in the U87MG-CTR group. Overexpression of *KCNB1* attenuated tumor growth by 65% when compared with the U87MG-CTR group (Fig. 5C). As shown in Fig. 5D, all xenograft glioma specimens were tested the protein levels of KCNB1. Western blot analysis showed that the protein levels of LC3II in xenografts from the U87MG-*KCNB1*+ group were much higher than those from the U87MG-CTR group (Fig. 5E), confirming that *KCNB1* overexpression induced autophagy *in vivo*.

Moreover, we injected U87MG-*KCNB1*+ or U87MG-CTR cells into the brains of nude mice. After 4 weeks of treatment, we monitored the size of tumors by MRI (Fig. 5F). All of the U87MG-*KCNB1*+ groups showed a significant decrease in tumor volumes (Fig. 5F). Kaplan–Meier survival analysis further showed that the U87MG-*KCNB1*+ group had a favorable prognosis (Fig. 5G).

## Discussion

Our results identified *KCNB1* as a novel prognostic indicator in gliomas and established its tumor suppressive activity as a modulator of apoptosis, autophagy, cell growth and invasion.

Genetic changes are a major contributing factor for glioma formation^29, 30^ and increasing evidence has shown that ion channel genes play an important role in the progression of gliomas^5, 8^. We have identified a correlation between the decreased expression of *KCNB1* and malignant progression of gliomas using three datasets of gliomas (CGGA, GSE16011, and REMBRANDT). In the present study, that contained 124 high-grade (III + IV) CGGA glioma samples, high *KCNB1* protein expression correlated with significantly lower OS and PFS, compared to patients with low *KCNB1* expression. These findings further support a tumor suppressor role for *KCNB1* in gliomas.

By what mechanism does *KCNB1* influence cancer processes? To test this, we used GO and KEGG pathway analysis of the differentially expressed genes in the *KCNB1* high expression group. GO analysis showed that the genes in this group were involved in apoptosis and cell death and KEGG pathway analysis showed that the MAPK signaling pathway was the predominant pathway in the high-*KCNB1*. Studies based on these biological analyses showed a significant increase in early and late apoptosis, accompanied by a marked accumulation of autophagic vacuoles, most likely via an ERK-dependent mechanism. The role of autophagy in glioma remains to be elucidated. The function of autophagy during tumor initiation or in established tumors can be highly distinct and context-dependent. Several investigations have shown that autophagy promotes tumorigenesis and survival cancers during hypoxia or nutrient starvation^31, 32^. Other studies suggested that autophagy promote apoptosis^33, 34^. Similarly, Magnolol and honokiol exert a synergistic anti-tumor effect through autophagy and apoptosis in human glioblastomas^35^.

While the role of ion gene channels for proliferation and apoptosis is largely recognized^5, 11, 19, 36–38^, the information regarding the molecular characteristics and mechanisms by which these channels regulate autophagy is still limited. Wang *et al.* ^39^ reported that cadmium (Cd^2+^) induces autophagy through elevation of cytosolic calcium via IP3R and subsequent extracellular signal-regulated kinase (ERK) activation. Overexpression of TRPML3, which provides Ca^2+^ that is required for fusion and fission events in autophagy, leads to increased autophagy in HeLa cells^40^. Our findings suggest that *KCNB1* may be an important regulator of autophagy via the ERK pathway.

The limitations of this study include the fact that *KCNB1* was tested retrospectively in gliomas. Further studies in prospective series and additional models are needed. However, our findings introduce the possibility that strategies aimed at restoring *KCNB1* activity may constitute a novel strategy that favors cancer cell death in specific subgroups of glioma patients.

In summary, our clinical data, together with our *in vitro* and *in vivo* findings, strongly suggest that gliomas are more aggressive if they have low expression of *KCNB1*. Moreover, *KCNB1* plays an important role in the induction of autophagy via activation of the ERK signaling pathway and could serve as a useful biomarker for the prognosis of patients with gliomas.

## Electronic supplementary material

Supplementary Information
