# Supplementary material for: Role of KCNB1 in the prognosis of gliomas and autophagy modulation
Source: Sci Rep. 2017 Feb 8;7:14. doi: 10.1038/s41598-017-00045-7 (PMC5428316; doi:10.1038/s41598-017-00045-7)

Title page

Role of KCNB1 in the prognosis of gliomas and autophagy modulation

Hao-Yuan Wang: Department of Neurosurgery, Zhujiang Hospital, Southern Medical University, Guangzhou, China, **email:**461251096@qq.com

Wen Wang: Department of Neurosurgery, Beijing Tiantan Hospital, Capital Medical University, Beijing, China, **email:** 675344815@qq.com

Yan-Wei Liu: Department of Neurosurgery, Beijing Tiantan Hospital, Capital Medical University, Beijing, China, **email:** liuyanwei_tiantan@163.com

Ming-Yang Li: Beijing Neurosurgical Institute, Capital Medical University, Beijing, China, **email:** 764988115@qq.com

Ting-Yu Liang: Beijing Neurosurgical Institute, Capital Medical University, Beijing, China, **email:** Liangtingyuttyy@126.com

Ji-Ye Li: Beijing Neurosurgical Institute, Capital Medical University, Beijing, China, **email:** lijiyettyy@126.com

Hui-Min Hu: Beijing Neurosurgical Institute, Capital Medical University, Beijing, China, **email:** huhuiminttyy@126.com

Yang Lu: Department of Neurosurgery, Zhujiang Hospital, Southern Medical University, Guangzhou, China, email: runminlyck@126.com

Chen Yao: Department of Neurosurgery, Zhujiang Hospital, Southern

Medical University, Guangzhou, China, email: 405570491@qq.com

Yong-Yi Ye: Department of Neurosurgery, Zhujiang Hospital, Southern Medical University, Guangzhou, China, email: 793834516@qq.com

Yong-Zhi Wang: Department of Neurosurgery, Beijing Tiantan Hospital, Capital Medical University, Beijing, China, **email:** wyz232@163.com

Shi-Zhong Zhang: Department of Neurosurgery, Zhujiang Hospital, Southern Medical University, Guangzhou, China, **email:** shizhong_zh@163.com

Running Title: Role of KCNB1 in the prognosis of gliomas

Competing interests

The authors declare no conflict of interest.

Total numbers of Figures and Tables: Five figures, one table, two supplementary figures and one supplementary table.

Correspondence Authors:

Yong-Zhi Wang: Department of Neurosurgery, Beijing Tiantan Hospital, Capital Medical University, No. 6 TiantanXili, Dongcheng District, Beijing 100050, China, Tel.: +86 10 67098431; fax: +86 10 67098431.

Shi-Zhong Zhang: Department of Neurosurgery, Zhujiang Hospital, Southern Medical University, 253# Gongye Road, Guangzhou, China, Tel.: +86 10 62782759; fax: +86 10 62782759.

Table S1. Ion channel genes involved in this study

Gene symbol	Gene title	Channel type
ANO1	anoctamin 1, Calcium activated chloride channel	Calcium activated chloride channel
ANO2	anoctamin 2, Calcium activated chloride channel	Calcium activated chloride channel
CACNA1A	calcium channel, voltage-dependent, P/Q type, alpha 1A subunit	Voltage-gated calcium channels
CACNA1B	calcium channel, voltage-dependent, N type, alpha 1B subunit	Voltage-gated calcium channels
CACNA1C	calcium channel, voltage-dependent, L type, alpha 1C subunit	Voltage-gated calcium channels
CACNA1D	calcium channel, voltage-dependent, L type, alpha 1D subunit	Voltage-gated calcium channels
CACNA1E	calcium channel, voltage-dependent, R type, alpha 1E subunit	Voltage-gated calcium channels
CACNA1F	calcium channel, voltage-dependent, L type, alpha 1F subunit	Voltage-gated calcium channels
CACNA1G	calcium channel, voltage-dependent, T type, alpha 1G subunit	Voltage-gated calcium channels
CACNA1H	calcium channel, voltage-dependent, T type, alpha 1H subunit	Voltage-gated calcium channels
CACNA1I	calcium channel, voltage-dependent, T type, alpha 1I subunit	Voltage-gated calcium channels
CACNA1S	calcium channel, voltage-dependent, L type, alpha 1S subunit	Voltage-gated calcium channels
CACNA2D1	calcium channel, voltage-dependent, alpha 2/delta subunit 1	Voltage-gated calcium channels
CACNA2D2	calcium channel, voltage-dependent, alpha 2/delta subunit 2	Voltage-gated calcium channels
CACNA2D3	calcium channel, voltage-dependent, alpha 2/delta subunit 3	Voltage-gated calcium channels
CACNA2D4	calcium channel, voltage-dependent, alpha 2/delta subunit 4	Voltage-gated calcium channels
CACNB1	calcium channel, voltage-dependent, beta 1 subunit	Voltage-gated calcium channels
CACNB2	calcium channel, voltage-dependent, beta 2 subunit	Voltage-gated calcium channels
CACNB3	calcium channel, voltage-dependent, beta 3 subunit	Voltage-gated calcium channels
CACNB4	calcium channel, voltage-dependent, beta 4 subunit	Voltage-gated calcium channels
CACNG1	calcium channel, voltage-dependent, gamma subunit 1	Voltage-gated calcium channels
CACNG2	calcium channel, voltage-dependent, gamma subunit 2	Voltage-gated calcium channels
CACNG3	calcium channel, voltage-dependent, gamma subunit 3	Voltage-gated calcium channels
CACNG4	calcium channel, voltage-dependent, gamma subunit 4	Voltage-gated calcium channels
CACNG5	calcium channel, voltage-dependent, gamma subunit 5	Voltage-gated calcium channels
CACNG6	calcium channel, voltage-dependent, gamma subunit 6	Voltage-gated calcium channels
CACNG7	calcium channel, voltage-dependent, gamma subunit 7	Voltage-gated calcium channels
CACNG8	calcium channel, voltage-dependent, gamma subunit 8	Voltage-gated calcium channels
CATSPER1	cation channel, sperm associated 1	CatSper and Two-Pore channels
CATSPER2	cation channel, sperm associated 2	CatSper and Two-Pore channels
CATSPER3	cation channel, sperm associated 3	CatSper and Two-Pore channels
CATSPER4	cation channel, sperm associated 4	CatSper and Two-Pore channels
CATSPERB	catsper channel auxiliary subunit beta	CatSper and Two-Pore channels
CATSPERD	catsper channel auxiliary subunit delta	CatSper and Two-Pore channels
CATSPERG	catsper channel auxiliary subunit gamma	CatSper and Two-Pore channels
CHRNA1	cholinergic receptor, nicotinic, alpha 1 (muscle)	Nicotinic acetylcholine receptors
CHRNA10	cholinergic receptor, nicotinic, alpha 10 (neuronal)	Nicotinic acetylcholine receptors
CHRNA2	cholinergic receptor, nicotinic, alpha 2 (neuronal)	Nicotinic acetylcholine receptors
CHRNA3	cholinergic receptor, nicotinic, alpha 3 (neuronal)	Nicotinic acetylcholine receptors
CHRNA4	cholinergic receptor, nicotinic, alpha 4 (neuronal)	Nicotinic acetylcholine receptors
CHRNA5	cholinergic receptor, nicotinic, alpha 5 (neuronal)	Nicotinic acetylcholine receptors

CHRNA6	cholinergic receptor, nicotinic, alpha 6 (neuronal)	Nicotinic acetylcholine receptors
CHRNA7	cholinergic receptor, nicotinic, alpha 7 (neuronal)	Nicotinic acetylcholine receptors
CHRNA9	cholinergic receptor, nicotinic, alpha 9 (neuronal)	Nicotinic acetylcholine receptors
CHRNB1	cholinergic receptor, nicotinic, beta 1 (muscle)	Nicotinic acetylcholine receptors
CHRNB2	cholinergic receptor, nicotinic, beta 2 (neuronal)	Nicotinic acetylcholine receptors
CHRNB3	cholinergic receptor, nicotinic, beta 3 (neuronal)	Nicotinic acetylcholine receptors
CHRNB4	cholinergic receptor, nicotinic, beta 4 (neuronal)	Nicotinic acetylcholine receptors
CHRND	cholinergic receptor, nicotinic, delta (muscle)	Nicotinic acetylcholine receptors
CHRNE	cholinergic receptor, nicotinic, epsilon (muscle)	Nicotinic acetylcholine receptors
CHRNG	cholinergic receptor, nicotinic, gamma (muscle)	Nicotinic acetylcholine receptors
CLCA1	chloride channel accessory 1	Calcium activated chloride channel
CLCA2	chloride channel accessory 2	Calcium activated chloride channel
CLCA3	chloride channel accessory 3	Calcium activated chloride channel
CLCC1	chloride channel CLIC-like 1	Mid-1-related chloride channel
CLCN1	chloride channel, voltage-sensitive 1	Voltage-sensitive chloride channel
CLCN2	chloride channel, voltage-sensitive 2	Voltage-sensitive chloride channel
CLCN3	chloride channel, voltage-sensitive 3	Voltage-sensitive chloride channel
CLCN4	chloride channel, voltage-sensitive 4	Voltage-sensitive chloride channel
CLCN5	chloride channel, voltage-sensitive 5	Voltage-sensitive chloride channel
CLCN6	chloride channel, voltage-sensitive 6	Voltage-sensitive chloride channel
CLCN7	chloride channel, voltage-sensitive 7	Voltage-sensitive chloride channel
CLCNKA	chloride channel, voltage-sensitive Ka	Voltage-sensitive chloride channel
CLCNKB	chloride channel, voltage-sensitive Kb	Voltage-sensitive chloride channel
CLIC1	chloride intracellular channel 1	Chloride intracellular channel
CLIC2	chloride intracellular channel 2	Chloride intracellular channel
CLIC3	chloride intracellular channel 3	Chloride intracellular channel
CLIC4	chloride intracellular channel 4	Chloride intracellular channel
CLIC5	chloride intracellular channel 5	Chloride intracellular channel
CLIC6	chloride intracellular channel 6	Chloride intracellular channel
CNGA1	cyclic nucleotide gated channel alpha 1	Cyclic nucleotide-regulated channels
CNGA2	cyclic nucleotide gated channel alpha 2	Cyclic nucleotide-regulated channels
CNGA3	cyclic nucleotide gated channel alpha 3	Cyclic nucleotide-regulated channels
CNGA4	cyclic nucleotide gated channel alpha 4	Cyclic nucleotide-regulated channels
CNGB1	cyclic nucleotide gated channel beta 1 C	yclic nucleotide-regulated channels
CNGB3	cyclic nucleotide gated channel beta 3 C	yclic nucleotide-regulated channels
GABARAP	GABA(A) receptor-associated protein	GABAA receptors
GABRA1	gamma-aminobutyric acid (GABA) A receptor, alpha 1	GABAA receptors
GABRA2	gamma-aminobutyric acid (GABA) A receptor, alpha 2	GABAA receptors
GABRA3	gamma-aminobutyric acid (GABA) A receptor, alpha 3	GABAA receptors
GABRA4	gamma-aminobutyric acid (GABA) A receptor, alpha 4	GABAA receptors
GABRA5	gamma-aminobutyric acid (GABA) A receptor, alpha 5	GABAA receptors
GABRA6	gamma-aminobutyric acid (GABA) A receptor, alpha 6	GABAA receptors
GABRB1	gamma-aminobutyric acid (GABA) A receptor, beta 1 G	ABAA receptors
GABRB2	gamma-aminobutyric acid (GABA) A receptor, beta 2 G	ABAA receptors

GABRB3	gamma-aminobutyric acid (GABA) A receptor, beta 3 G	ABAA receptors
GABRD	gamma-aminobutyric acid (GABA) A receptor, delta GA	BAA receptors
GABRE	gamma-aminobutyric acid (GABA) A receptor, epsilon	GABAA receptors
GABRG1	gamma-aminobutyric acid (GABA) A receptor, gamma 1	GABAA receptors
GABRG2	gamma-aminobutyric acid (GABA) A receptor, gamma 2	GABAA receptors
GABRG3	gamma-aminobutyric acid (GABA) A receptor, gamma 3	GABAA receptors
GABRP	gamma-aminobutyric acid (GABA) A receptor, pi	GABAA receptors
GABRQ	gamma-aminobutyric acid (GABA) A receptor, theta	GABAA receptors
GABRR1	gamma-aminobutyric acid (GABA) A receptor, rho 1	GABAA receptors
GABRR2	gamma-aminobutyric acid (GABA) A receptor, rho 2	GABAA receptors
GABRR3	gamma-aminobutyric acid (GABA) A receptor, rho 3	GABAA receptors
GLRA1	glycine receptor, alpha 1	Glycine receptors
GLRA2	glycine receptor, alpha 2	Glycine receptors
GLRA3	glycine receptor, alpha 3	Glycine receptors
GLRA4	glycine receptor, alpha 4	Glycine receptors
GLRB	glycine receptor, beta	Glycine receptors
GRIA1	glutamate receptor, ionotropic, AMPA 1	Ionotropic glutamate receptors
GRIA2	glutamate receptor, ionotropic, AMPA 2	Ionotropic glutamate receptors
GRIA3	glutamate receptor, ionotropic, AMPA 3	Ionotropic glutamate receptors
GRIA4	glutamate receptor, ionotropic, AMPA 4	Ionotropic glutamate receptors
GRID1	glutamate receptor, ionotropic, delta 1	Ionotropic glutamate receptors
GRID2	glutamate receptor, ionotropic, delta 2	Ionotropic glutamate receptors
GRIK1	glutamate receptor, ionotropic, kainate 1	Ionotropic glutamate receptors
GRIK2	glutamate receptor, ionotropic, kainate 2	Ionotropic glutamate receptors
GRIK3	glutamate receptor, ionotropic, kainate 3	Ionotropic glutamate receptors
GRIK4	glutamate receptor, ionotropic, kainate 4	Ionotropic glutamate receptors
GRIK5	glutamate receptor, ionotropic, kainate 5	Ionotropic glutamate receptors
GRIN1	glutamate receptor, ionotropic, N-methyl D-aspartate 1	Ionotropic glutamate receptors
GRIN2A	glutamate receptor, ionotropic, N-methyl D-aspartate 2A	Ionotropic glutamate receptors
GRIN2B	glutamate receptor, ionotropic, N-methyl D-aspartate 2B	Ionotropic glutamate receptors
GRIN2C	glutamate receptor, ionotropic, N-methyl D-aspartate 2C	Ionotropic glutamate receptors
GRIN2D	glutamate receptor, ionotropic, N-methyl D-aspartate 2D	Ionotropic glutamate receptors
GRIN3A	glutamate receptor, ionotropic, N-methyl-D-aspartate 3A	Ionotropic glutamate receptors
GRIN3B	glutamate receptor, ionotropic, N-methyl-D-aspartate 3B	Ionotropic glutamate receptors
HCN1	hyperpolarization activated cyclic nucleotide-gated potassium channel 1	Cyclic nucleotide-regulated channels
HCN2	hyperpolarization activated cyclic nucleotide-gated potassium channel 1	Cyclic nucleotide-regulated channels
HCN3	hyperpolarization activated cyclic nucleotide-gated potassium channel 1	Cyclic nucleotide-regulated channels
HCN4	hyperpolarization activated cyclic nucleotide-gated potassium channel 1	Cyclic nucleotide-regulated channels
HTR3A	5-hydroxytryptamine (serotonin) receptor 3A,	ionotropic 5-HT3 receptors
HTR3B	5-hydroxytryptamine (serotonin) receptor 3B,	ionotropic 5-HT3 receptors
HTR3C	5-hydroxytryptamine (serotonin) receptor 3C,	ionotropic 5-HT3 receptors
HTR3D	5-hydroxytryptamine (serotonin) receptor 3D,	ionotropic 5-HT3 receptors
HTR3E	5-hydroxytryptamine (serotonin) receptor 3E,	ionotropic 5-HT3 receptors
HVCN1	hydrogen voltage-gated channel 1	Voltage-gated proton channel

KCNA1	potassium voltage-gated channel, shaker-related subfamily, member 1 (episodic ataxia with myokymia)	Voltage-gated potassium channels
KCNA10	potassium voltage-gated channel, shaker-related subfamily, member 10 (episodic ataxia with myokymia)	Voltage-gated potassium channels
KCNA2	potassium voltage-gated channel, shaker-related subfamily, member 2 (episodic ataxia with myokymia)	Voltage-gated potassium channels
KCNA3	potassium voltage-gated channel, shaker-related subfamily, member 3 (episodic ataxia with myokymia)	Voltage-gated potassium channels
KCNA4	potassium voltage-gated channel, shaker-related subfamily, member 4 (episodic ataxia with myokymia)	Voltage-gated potassium channels
KCNA5	potassium voltage-gated channel, shaker-related subfamily, member 5 (episodic ataxia with myokymia)	Voltage-gated potassium channels
KCNA6	potassium voltage-gated channel, shaker-related subfamily, member 6 (episodic ataxia with myokymia)	Voltage-gated potassium channels
KCNA7	potassium voltage-gated channel, shaker-related subfamily, member 7 (episodic ataxia with myokymia)	Voltage-gated potassium channels
KCNAB1	potassium voltage-gated channel, shaker-related subfamily, beta member 1	Voltage-gated potassium channels
KCNAB2	potassium voltage-gated channel, shaker-related subfamily, beta member 2	Voltage-gated potassium channels
KCNAB3	potassium voltage-gated channel, shaker-related subfamily, beta member 3	Voltage-gated potassium channels
KCNB1	potassium voltage-gated channel, Shab-related subfamily, member 1	Voltage-gated potassium channels
KCNB2	potassium voltage-gated channel, Shab-related subfamily, member 2	Voltage-gated potassium channels
KCNC1	potassium voltage-gated channel, Shaw-related subfamily, member 1	Voltage-gated potassium channels
KCNC2	potassium voltage-gated channel, Shaw-related subfamily, member 2	Voltage-gated potassium channels
KCNC3	potassium voltage-gated channel, Shaw-related subfamily, member 3	Voltage-gated potassium channels
KCNC4	potassium voltage-gated channel, Shaw-related subfamily, member 4	Voltage-gated potassium channels
KCND1	potassium voltage-gated channel, Shal-related subfamily, member 1	Voltage-gated potassium channels
KCND2	potassium voltage-gated channel, Shal-related subfamily, member 2	Voltage-gated potassium channels
KCND3	potassium voltage-gated channel, Shal-related subfamily, member 3	Voltage-gated potassium channels
KCNE1	potassium voltage-gated channel, Isk-related family, member 1	Voltage-gated potassium channels
KCNE1L	KCNE1-like	Voltage-gated potassium channels
KCNE2	potassium voltage-gated channel, Isk-related family, member 2	Voltage-gated potassium channels
KCNE3	potassium voltage-gated channel, Isk-related family, member 3	Voltage-gated potassium channels
KCNE4	potassium voltage-gated channel, Isk-related family, member 4	Voltage-gated potassium channels
KCNF1	potassium voltage-gated channel, subfamily F, member 1	Voltage-gated potassium channels
KCNG1	potassium voltage-gated channel, subfamily G, member 1	Voltage-gated potassium channels
KCNG2	potassium voltage-gated channel, subfamily G, member 2	Voltage-gated potassium channels
KCNG3	potassium voltage-gated channel, subfamily G, member 3	Voltage-gated potassium channels
KCNG4	potassium voltage-gated channel, subfamily G, member 4	Voltage-gated potassium channels
KCNH1	potassium voltage-gated channel, subfamily H (eagrelated), member 1	Voltage-gated potassium channels
KCNH2	potassium voltage-gated channel, subfamily H (eagrelated), member 2	Voltage-gated potassium channels
KCNH3	potassium voltage-gated channel, subfamily H (eagrelated), member 3	Voltage-gated potassium channels
KCNH4	potassium voltage-gated channel, subfamily H (eagrelated), member 4	Voltage-gated potassium channels
KCNH5	potassium voltage-gated channel, subfamily H (eagrelated), member 5	Voltage-gated potassium channels
KCNH6	potassium voltage-gated channel, subfamily H (eagrelated), member 6	Voltage-gated potassium channels

KCNH7	potassium voltage-gated channel, subfamily H (eagrelated), member 7	Voltage-gated potassium channels
KCNH8	potassium voltage-gated channel, subfamily H (eagrelated), member 8	Voltage-gated potassium channels
KCNJ1	potassium inwardly-rectifying channel, subfamily J, member 1	Inwardly rectifying potassium channels
KCNJ10	potassium inwardly-rectifying channel, subfamily J, member 10	Inwardly rectifying potassium channels
KCNJ11	potassium inwardly-rectifying channel, subfamily J, member 11	Inwardly rectifying potassium channels
KCNJ12	potassium inwardly-rectifying channel, subfamily J, member 12	Inwardly rectifying potassium channels
KCNJ13	potassium inwardly-rectifying channel, subfamily J, member 13	Inwardly rectifying potassium channels
KCNJ14	potassium inwardly-rectifying channel, subfamily J, member 14	Inwardly rectifying potassium channels
KCNJ15	potassium inwardly-rectifying channel, subfamily J, member 15	Inwardly rectifying potassium channels
KCNJ16	potassium inwardly-rectifying channel, subfamily J, member 16	Inwardly rectifying potassium channels
KCNJ18	potassium inwardly-rectifying channel, subfamily J, member 18	Inwardly rectifying potassium channels
KCNJ2	potassium inwardly-rectifying channel, subfamily J, member 2	Inwardly rectifying potassium channels
KCNJ3	potassium inwardly-rectifying channel, subfamily J, member 3	Inwardly rectifying potassium channels
KCNJ4	potassium inwardly-rectifying channel, subfamily J, member 4	Inwardly rectifying potassium channels
KCNJ5	potassium inwardly-rectifying channel, subfamily J, member 5	Inwardly rectifying potassium channels
KCNJ6	potassium inwardly-rectifying channel, subfamily J, member 6	Inwardly rectifying potassium channels
KCNJ8	potassium inwardly-rectifying channel, subfamily J, member 8	Inwardly rectifying potassium channels
KCNJ9	potassium inwardly-rectifying channel, subfamily J, member 9	Inwardly rectifying potassium channels
KCNK1	potassium channel, subfamily K, member 1	Two-P potassium channels
KCNK10	potassium channel, subfamily K, member 10	Two-P potassium channels
KCNK12	potassium channel, subfamily K, member 12	Two-P potassium channels
KCNK13	potassium channel, subfamily K, member 13	Two-P potassium channels
KCNK15	potassium channel, subfamily K, member 15	Two-P potassium channels
KCNK16	potassium channel, subfamily K, member 16	Two-P potassium channels
KCNK17	potassium channel, subfamily K, member 17	Two-P potassium channels
KCNK18	potassium channel, subfamily K, member 18	Two-P potassium channels
KCNK2	potassium channel, subfamily K, member 2	Two-P potassium channels
KCNK3	potassium channel, subfamily K, member 3	Two-P potassium channels
KCNK4	potassium channel, subfamily K, member 4	Two-P potassium channels
KCNK5	potassium channel, subfamily K, member 5	Two-P potassium channels
KCNK6	potassium channel, subfamily K, member 6	Two-P potassium channels
KCNK7	potassium channel, subfamily K, member 7	Two-P potassium channels
KCNK9	potassium channel, subfamily K, member 9	Two-P potassium channels
KCNMA1	potassium large conductance calcium-activated channel, subfamily M, alpha member 1	Calcium-activated potassium channels
KCNMB1	potassium large conductance calcium-activated channel, subfamily M, beta member 1	Calcium-activated potassium channels
KCNMB2	potassium large conductance calcium-activated channel, subfamily M, beta member 2	Calcium-activated potassium channels
KCNMB3	potassium large conductance calcium-activated channel, subfamily M, beta member 3	Calcium-activated potassium channels
KCNMB4	potassium large conductance calcium-activated channel, subfamily M, beta member 4	Calcium-activated potassium channels
KCNN1	potassium intermediate/small conductance calciumactivated channel, subfamily N, member 1	Calcium-activated potassium channels
KCNN2	potassium intermediate/small conductance calciumactivated channel, subfamily N, member 2	Calcium-activated potassium channels
KCNN3	potassium intermediate/small conductance calciumactivated channel, subfamily N, member 3	Calcium-activated potassium channels

KCNN4	potassium intermediate/small conductance calciumactivated channel, subfamily N, member 4	Calcium-activated potassium channels
KCNQ1	potassium voltage-gated channel, KQT-like subfamily, member 1	Voltage-gated potassium channels
KCNQ2	potassium voltage-gated channel, KQT-like subfamily, member 2	Voltage-gated potassium channels
KCNQ3	potassium voltage-gated channel, KQT-like subfamily, member 3	Voltage-gated potassium channels
KCNQ4	potassium voltage-gated channel, KQT-like subfamily, member 4	Voltage-gated potassium channels
KCNQ5	potassium voltage-gated channel, KQT-like subfamily, member 5	Voltage-gated potassium channels
KCNS1	potassium voltage-gated channel, delayed-rectifier, subfamily S, member 1	Voltage-gated potassium channels
KCNS2	potassium voltage-gated channel, delayed-rectifier, subfamily S, member 2	Voltage-gated potassium channels
KCNS3	potassium voltage-gated channel, delayed-rectifier, subfamily S, member 3	Voltage-gated potassium channels
KCNT1	potassium channel, subfamily T, member 1	Calcium-activated potassium channels
KCNT2	potassium channel, subfamily T, member 2	Calcium-activated potassium channels
KCNU1	potassium channel, subfamily U, member 1	Calcium-activated potassium channels
KCNV1	potassium channel, subfamily V, member 1	Voltage-gated potassium channels
KCNV2	potassium channel, subfamily V, member 2	Voltage-gated potassium channels
MCOLN1	mucolipin 1	Transient receptor potential channels
MCOLN2	mucolipin 2	Transient receptor potential channels
MCOLN3	mucolipin 3	Transient receptor potential channels
NALCN	sodium leak channel, non-selective	Voltage-independent cation channels
P2RX1	purinergic receptor P2X, ligand-gated ion channel, 1	P2X receptors
P2RX2	purinergic receptor P2X, ligand-gated ion channel, 2	P2X receptors
P2RX3	purinergic receptor P2X, ligand-gated ion channel, 3	P2X receptors
P2RX4	purinergic receptor P2X, ligand-gated ion channel, 4	P2X receptors
P2RX5	purinergic receptor P2X, ligand-gated ion channel, 5	P2X receptors
P2RX6	purinergic receptor P2X, ligand-gated ion channel, 6	P2X receptors
P2RX7	purinergic receptor P2X, ligand-gated ion channel, 7	P2X receptors
PKD1	polycystic kidney disease 1 (autosomal dominant)	Transient receptor potential channels
PKD2	polycystic kidney disease 2 (autosomal dominant)	Transient receptor potential channels
PKD2L1	polycystic kidney disease 2-like 1	Transient receptor potential channels
PKD2L2	polycystic kidney disease 2-like 2	Transient receptor potential channels
SCN10A	sodium channel, voltage-gated, type X, alpha subunit	Voltage-gated sodium channels
SCN11A	sodium channel, voltage-gated, type XI, alpha subunit	Voltage-gated sodium channels
SCN1A	sodium channel, voltage-gated, type I, alpha subunit	Voltage-gated sodium channels
SCN1B	sodium channel, voltage-gated, type I, beta subunit	Voltage-gated sodium channels
SCN2A	sodium channel, voltage-gated, type II, alpha subunit	Voltage-gated sodium channels
SCN2B	sodium channel, voltage-gated, type II, beta subunit	Voltage-gated sodium channels
SCN3A	sodium channel, voltage-gated, type III, alpha subunit	Voltage-gated sodium channels
SCN3B	sodium channel, voltage-gated, type III, beta subunit	Voltage-gated sodium channels
SCN4A	sodium channel, voltage-gated, type IV, alpha subunit	Voltage-gated sodium channels
SCN4B	sodium channel, voltage-gated, type IV, beta subunit	Voltage-gated sodium channels
SCN5A	sodium channel, voltage-gated, type V, alpha subunit	Voltage-gated sodium channels
SCN7A	sodium channel, voltage-gated, type VII, alpha subunit	Voltage-gated sodium channels
SCN8A	sodium channel, voltage gated, type VIII, alpha subunit	Voltage-gated sodium channels
SCN9A	sodium channel, voltage-gated, type IX, alpha subunit	Voltage-gated sodium channels

SCNN1A	sodium channel, non-voltage-gated 1 alpha subunit	Nonvoltage-gated sodium channels
SCNN1B	sodium channel, non-voltage-gated 1, beta subunit	Nonvoltage-gated sodium channels
SCNN1D	sodium channel, non-voltage-gated 1, delta subunit	Nonvoltage-gated sodium channels
SCNN1G	sodium channel, non-voltage-gated 1, gamma subunit	Nonvoltage-gated sodium channels
TPCN1	two pore segment channel 1	CatSper and two-pore channels
TPCN2	two pore segment channel 2	CatSper and two-pore channels
TRPA1	transient receptor potential cation channel, subfamily A, member 1	Transient receptor potential channels
TRPC1	transient receptor potential cation channel, subfamily C, member 1	Transient receptor potential channels
TRPC3	transient receptor potential cation channel, subfamily C, member 3	Transient receptor potential channels
TRPC4	transient receptor potential cation channel, subfamily C, member 4	Transient receptor potential channels
TRPC5	transient receptor potential cation channel, subfamily C, member 5	Transient receptor potential channels
TRPC6	transient receptor potential cation channel, subfamily C, member 6	Transient receptor potential channels
TRPC7	transient receptor potential cation channel, subfamily C, member 7	Transient receptor potential channels
TRPM1	transient receptor potential cation channel, subfamily M, member 1	Transient receptor potential channels
TRPM2	transient receptor potential cation channel, subfamily M, member 2	Transient receptor potential channels
TRPM3	transient receptor potential cation channel, subfamily M, member 3	Transient receptor potential channels
TRPM4	transient receptor potential cation channel, subfamily M, member 4	Transient receptor potential channels
TRPM5	transient receptor potential cation channel, subfamily M, member 5	Transient receptor potential channels
TRPM6	transient receptor potential cation channel, subfamily M, member 6	Transient receptor potential channels
TRPM7	transient receptor potential cation channel, subfamily M, member 7	Transient receptor potential channels
TRPM8	transient receptor potential cation channel, subfamily M, member 8	Transient receptor potential channels
TRPV1	transient receptor potential cation channel, subfamily V, member 1	Transient receptor potential channels
TRPV2	transient receptor potential cation channel, subfamily V, member 2	Transient receptor potential channels
TRPV3	transient receptor potential cation channel, subfamily V, member 3	Transient receptor potential channels
TRPV4	transient receptor potential cation channel, subfamily V, member 4	Transient receptor potential channels
TRPV5	transient receptor potential cation channel, subfamily V, member 5	Transient receptor potential channels
TRPV6	transient receptor potential cation channel, subfamily V, member 6	Transient receptor potential channels
VDAC1	voltage-dependent anion channel 1	voltage-dependent anion channel
VDAC2	voltage-dependent anion channel 2	voltage-dependent anion channel
VDAC3	voltage-dependent anion channel 3	voltage-dependent anion channel
ZACN	zinc activated ligand-gated ion channel	ZAC

Figure S1. EX-T1882-Lv201 vectors for the transduction of the KCNB1 gene.

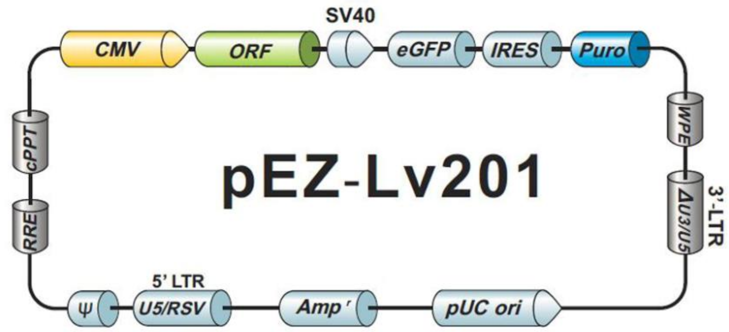

Figure S2. (**A**) Immunofluorescence of KCNB1+ (eGFP) cells. (**B**) Modulation of migration in U87MG and U118MG cells by KCNB1 transduction and its rescue by specific KCNB1 siRNA. *P* values were calculated with two-sided Student's t test. Scale bar, 25um. * $p < 0.05$.

A

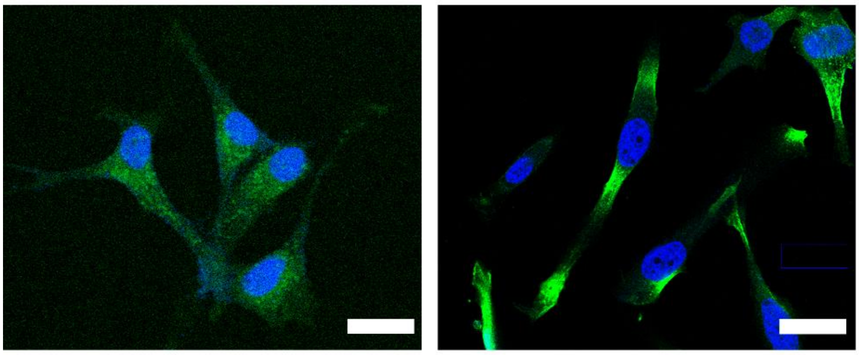

B

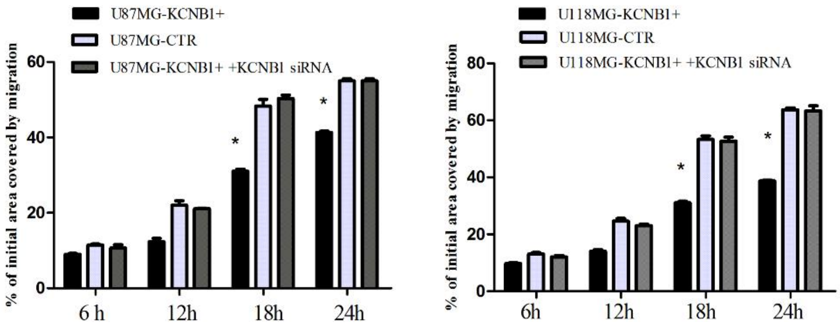

Supplement: Supplementary file 1 — Supplementary Information [file 41598_2017_45_MOESM1_ESM.pdf]
